# Cystic Interstitial Lung Diseases: A Pictorial Review and a Practical Guide for the Radiologist

**DOI:** 10.3390/diagnostics10060346

**Published:** 2020-05-27

**Authors:** Giulia Aquilina, Daniele Carmelo Caltabiano, Federica Galioto, Giovanna Cancemi, Fabio Pino, Ada Vancheri, Carlo Vancheri, Pietro Valerio Foti, Letizia Antonella Mauro, Antonio Basile

**Affiliations:** 1 Department of Medical Surgical Sciences and Advanced Technologies “GF Ingrassia”—Radiology Unit I. University hospital “Policlinico-Vittorio Emanuele” Via Santa Sofia 78, 95123 Catania, Italy; federicagalioto91@gmail.com (F.G.); cancemi.giovanna@gmail.com (G.C.); pietrofoti@hotmail.com (P.V.F.); basile.antonello73@gmail.com (A.B.); 2 Department of Diagnostic Radiology “Umberto I” Hospital, Contrada Ferrante, 94100 Enna, Italy; daniele.788@gmail.com; 3 Department of Clinical and Experimental Medicine, University of Catania, Regional Referral Centre for Rare Lung Disease, 95123 Catania, Italy; pinofabio@outlook.it (F.P.); adact@hotmail.it (A.V.); vancheri@unict.it (C.V.)

**Keywords:** lung, multidetector computed tomography, cyst, lung disease, interstitial

## Abstract

A cyst is a round circumscribed area of low attenuation, surrounded by epithelial or fibrous wall. Cysts can frequently occur on chest computed tomography (CT) and high-resolution computed tomography (HRCT); multiple parenchymal cysts of the lungs are the most typical feature of cystic lung interstitial diseases, characterizing a wide spectrum of diseases—ranging from isolated lung disorders up to diffuse pulmonary diseases. The aim of this review is to analyze scientific literature about cystic lung interstitial diseases and to provide a practical guide for radiologists, focusing on the main morphological features of pulmonary cysts: size, shape, borders, wall, location, and distribution. These features are shown on free-hand drawings and related to HRCT images, in order to help radiologists pursue the correct differential diagnosis between similar conditions.

## 1. Introduction

A cystic pulmonary lesion is a round circumscribed, well-defined air-containing space which refers—more in detail—to the presence of an area of low density of 1 centimeter or more in diameter [1,2]. Cysts may be seen in patients with different pulmonary disorders, such as honeycombing, pneumonia (as pneumatoceles), and cystic bronchiectases (permanent dilatation of bronchi); they may be also associated with lungs of the elderly, or they may also be related to trauma with laceration or parasitic infections. In addition, they may be the main location of several lung rare diseases—such as Langerhans cell histiocytosis (LCH), lymphangioleiomyomatosis (LAM), lymphocytic interstitial pneumonitis (LIP), desquamative interstitial pneumonitis (DIP), neurofibromatosis (NF1) and Birt–Hogg–Dubè (BHD) syndrome [3,4,5]. The diagnosis should be based on the number, shape, size, morphology and distribution of cysts within the lung; it should be focused also on the possible detection of associated lung parenchymal abnormalities and should always be related to the clinical history of the patient, in order to provide the most helpful diagnostic clues for diagnosing specific cystic lung diseases.

This review aims to be a practical and helpful guide for radiologists and non-radiologists describing the main findings in cystic lung diseases and pursuing the correct differential diagnosis among similar conditions. We emphasize the main high-resolution computed tomography (HRCT) features of the diseases already described, providing an accurate description of the morphological characteristics of these cystic lesions. This knowledge of cystic CT patterns—even if heterogeneous and various—is crucial to identify the correct disease and plan the appropriate treatment; although a multidisciplinary approach is necessary to make the correct diagnosis, a radiologic approach is important for narrowing the differential diagnosis. Therefore, we strongly stress the importance for these patients to undergo a CT examination; still, a definite diagnosis may require clinical correlation and, occasionally, biopsy.

## 2. Materials and Methods

We extensively searched on Med-Line database for articles in the English language from highly-impacted journals, using the key-words “cysts” AND “interstitial” AND “lung” AND “diseases” AND “imaging” and examined titles and abstracts; we excluded irrelevant articles, recurrent articles from the same author, non-English articles, articles not related to the topic, and articles which we could not access the entire content of. We also included articles which were obtained from the reference lists of the retrieved articles. We finally selected 68 articles, as shown in a flow chart (Supplementary Materials), from which we deduced the results we have stated. Then, we examined chest HRCT scans performed in our radiology unit between 2016 and 2020, searching in our databases using as keywords “cysts” OR “Langerhans Cell Histiocytosis” OR “Lymphangioleiomyomatosis”, OR “Lymphocytic interstitial pneumonitis” OR “Neurofibromatosis” OR “Desquamative Interstitial Pneumoniae” OR “Birt–Hogg–Dubè Syndrome”, and look for significant cases with parenchymal cysts. Then, we made some free-hand drawings to show reproduce the main morphological characteristics of cystic alterations that occur in these conditions; each drawing is referred to the real case image.

## 3. Results

We have analyzed 68 articles using the stated criteria; we have assumed that lung cysts may be classified according to (1) the wall thickness (thin or thick), (2) anatomical distribution (spread or sparing of particular areas of the lung), (3) number, (4) morphology, (5) clinical manifestation (infectious and non-infectious disease) and (6) clinical-anamnestic information (age, gender and smoking habits). The main radiological features have been summarized in Table 1. An accurate description of these features is mandatory to address the diagnosis, identifying the correct interstitial lung disease. 

## 4. Discussion

On computed tomography (CT) scans, a cyst is described as a small round area of hyperlucency or low parenchymal attenuation, having well-defined interfaces—surrounded by the normal lung parenchyma; cysts may have a variable wall thickness, although they are usually characterized by a thin wall (<2 mm) and they occur in the absence of emphysema. Their content is predominantly air, even if sometimes they may contain fluid or corpuscular material [5]. Cystic lesions are supposed to have different pathogenic mechanisms: they may origin by a “valve” mechanism at bronchiolar level—as we can observe in LIP—or may be caused by parenchymal destruction, fibrotic traction or nodule excavation—as we can see in histiocytosis.

### 4.1. Histiocytosis X or Pulmonary Langerhans Cell Histiocytosis (LCH)

LCH is part of a group of diseases—often diagnosed during childhood; in this spectrum of conditions, Langerhans cells accumulation affects many organs, such as lung, bone, pituitary gland, skin and liver; the lung is the most frequently affected organ (up to 40% of patients) and may occur as an isolated manifestation.

LCH affects mostly young or middle-aged adults (aged between 20 and 40 years), having a slight predominance in the male gender [3]. Cigarette smoking, including passive smoking, could be a determining factor in the accumulation and activation of these cells [4], through the activation of cytokines such as Granulocyte Macrophage-Colony-Stimulating Factor (GM-CSF) and Transforming growth factor β (TGF β). The main clinical findings are represented by cough, dyspnea and fatigue; in 20% of patients, the first manifestation can be pneumothorax [5,6]; less frequently, it manifests with chest pain and fever [7]. Diagnosis of LCH should be taken into account in patients with a positive history of skin rash and diabetes insipidus. 

In the early phase of this disease, the lung parenchyma shows the presence of starry granulomatous nodules, arising from the massive proliferation of peri-bronchiolar infiltrates of Langerhans cells and eosinophils, which are regulators of the mucosal immunity of the airways, resulting in destruction of lung tissue [8]. In the later stages, the increasing of the central air component in these nodules, associated with developing of a certain grade of fibrosis, determines the cysts’ production. 

**Figure 1:**
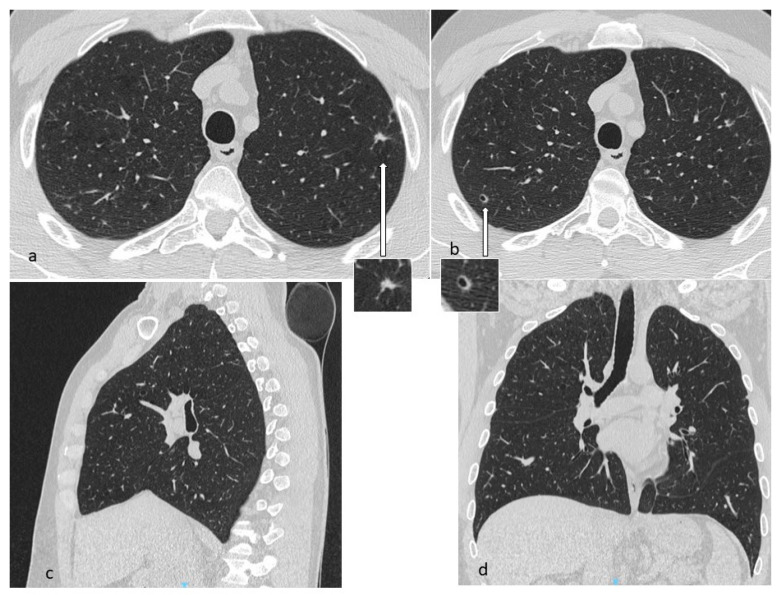
In LCH, cysts may origin from the cavitation of nodules (**a**,**b**) and can be isolated or confluent, and are mainly located in the middle and superior lobes, with typical sparing of costophrenic angles. Multiplanar reconstruction (MPR) in the sagittal (**c**) and coronal plane (**d**).

Morphologically, these bronchioles-centric lesions evolve from micro- and macro-nodules—with a highly represented cellular component—to lesions with low cellularity, often star-shaped, with fibrotic scar [9]. In the advanced stages of the disease, the nodule’s cavitation is the most common evolution—resembling a cystic appearance (Figure 1).

**Figure 2:**
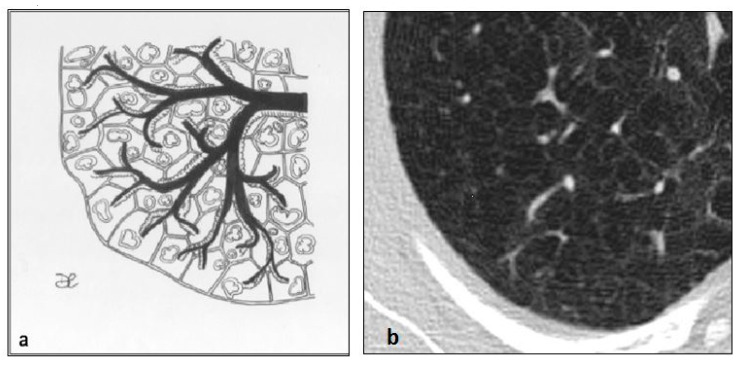
LCH cystic pattern (**a**). Lung cysts, ranging from 10 to 20 millimeters of diameter, are depicted in figures (**a**) and (**b**). These cysts show thick walls and bizarre shape.

**Figure 3:**
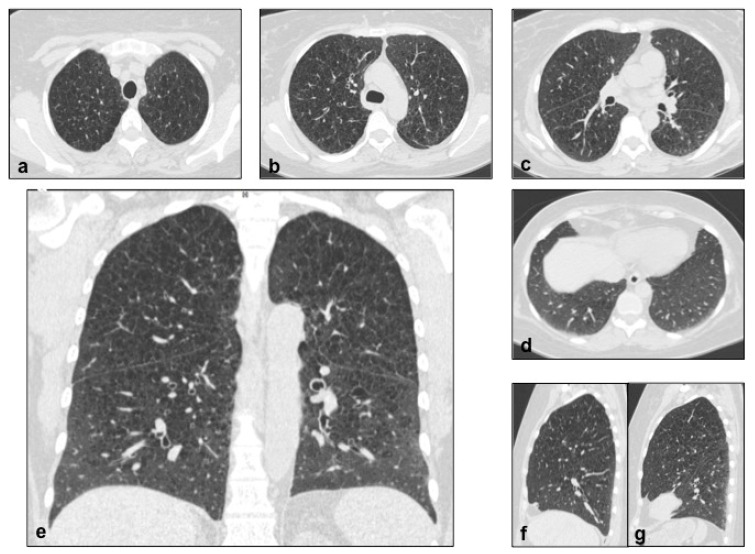
LCH cystic pattern, with multiple cysts predominantly located in the upper lobes (**a**,**b**) and in the apical segments of the lower lobes (**c**), with typical sparing of costophrenic angles (**d**). On multiplanar reconstructions (MPR) in the coronal (**e**) and sagittal (**f**,**g**) plane is evident the typical distribution of lesions. This figure has been partially modified from ECR 2017/C-2141 Cystic pattern in lung diseases: a simplified HRCT guide based on free-hand drawings, DC Caltabiano, V. Costanzo, L. Mammino, V. Vindigni, S. Torrisi, R. Rosso, LA Mauro, C. Vancheri, S. Palmucci.

**Figure 4:**
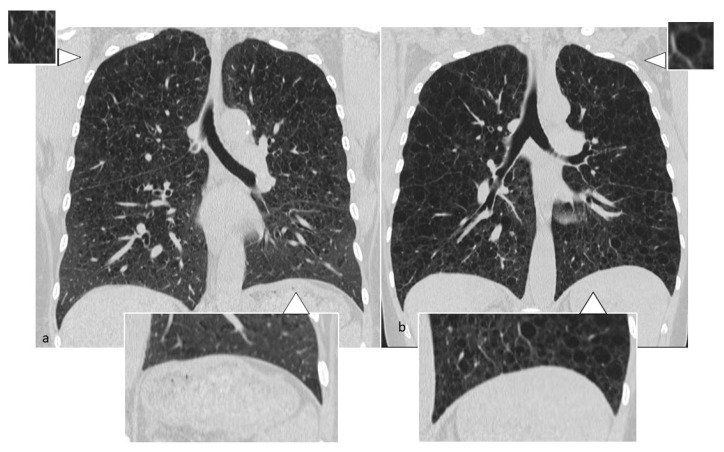
Differential diagnosis between LCH and LAM: in LCH (**a**), cysts present bizarre shape, polilobular contours and thick walls, with sparing of costophrenic angles. In LAM (**b**), cysts are regular and rounded, uniform in size, with thin walls; they have wide distribution, including costophrenic angles.

Lung cysts, which are the most advanced expression of the evolution of LCH, may vary in size from one to several centimeters; they occur with thick wall and “bizarre” morphology, having poli-lobulated or clover-leaf contours, in contrast to the more uniform and bland cysts typical of other diffuse cystic lung diseases, such as LAM; the cysts have also a wide distribution (Figure 2), with a predominance in the lung apices and the medium regions, typically sparing costophrenic angles (Figure 3); this distribution is one of the diagnostic keys that distinguishes LCH from LAM [10] (Figure 4). Another helpful clue is the sparing of the medial tips of medial lobe and lingula; lung volumes are normal or increased, an unusual appearance when reticular opacities or honeycombing occur. Frequently, cysts are the only alteration found in HRCT scans, but in most cases small nodules are also detectable.

The course of the disease is variable: in cases having an early diagnosis—without pulmonary cysts—smoking cessation may produce spontaneously nodular lesions regression; cystic lesions, once formed, persist because they represent an irreversible damage. In the advanced phase, the disease may develop into a fibrotic pattern, showing bizarre shaped cysts, reticulations and honeycombing [11].

Normal values of lung function tests or a restrictive framework are appreciable in the early stages of LCH, while an obstructive framework predominates in the advanced stages [12]. Bronchoscopy with transbronchial lung biopsy is useful to perform diagnosis in 30% of cases, but in the remaining cases surgical lung biopsy is necessary for definite diagnosis [13]. 

### 4.2. Lymphangioleiomiomatosis (LAM)

LAM is a rare, multi-organ disease, characterized by progressive proliferation of immature smooth muscle cells (LAM cells), that arise from an unknown source and spread via blood and lymphatic vessels in the airways, in the chest and in the abdomen. An association with tuberous sclerosis (TSC-LAM complex) is known, with mutations of two known genes (*TSC1* and *TSC2*) found in these cases. The “sporadic” form is observed in patients who do not have tuberous sclerosis (S-LAM), having only *TSC2* gene mutation [14]. In patients affected by TSC-LAM, the mutations of two genes are present in all the lineages of cells, including the germline, which occasionally determines vertical transmission; instead, in patients with S-LAM, mutations appear after conception in confined tissues, such as the kidney, lung and lymph nodes, in the absence of vertical transmission [15]. *TSC1* and *TSC2* encode two proteins, called hamartin and tuberin, which together form a heterodimeric protein responsible for cell growth and cellular survival; their genic mutation determines inappropriate cellular proliferation. 

**Figure 5:**
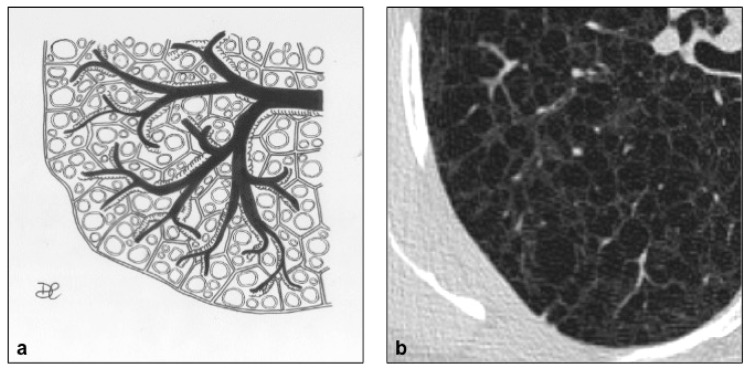
LAM cystic pattern (**a**). Lung cysts with rounded regular shape and thin walls are shown on pictures (**a**,**b**). This figure has been partially modified from ECR 2017/C-2141 Cystic pattern in lung diseases: a simplified HRCT guide based on free-hand drawings, DC Caltabiano, V. Costanzo, L. Mammino, V. Vindigni, S. Torrisi, R. Rosso, LA Mauro, C. Vancheri, S. Palmucci.

**Figure 6:**
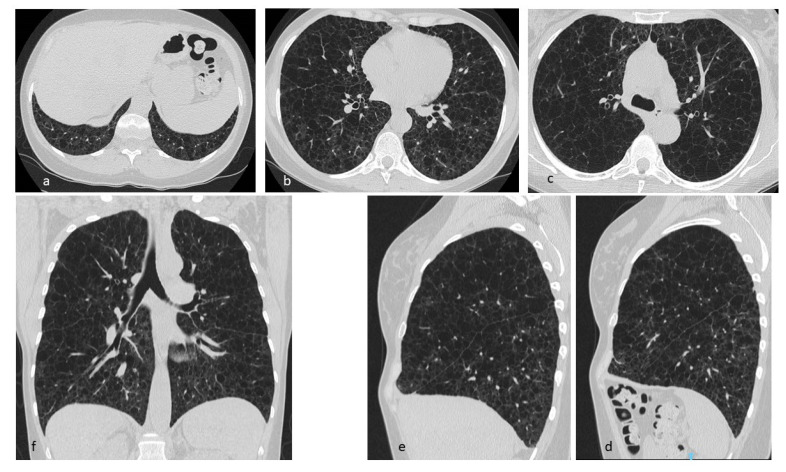
LAM: multiple cysts in pulmonary parenchyma with diffuse distribution (**a**–**c**), including apices, basal regions and costophrenic angles (**d**). Multiplanar reconstructions (MPR) in the sagittal (**d**,**e**) and coronal (**f**) plane.

**Figure 7:**
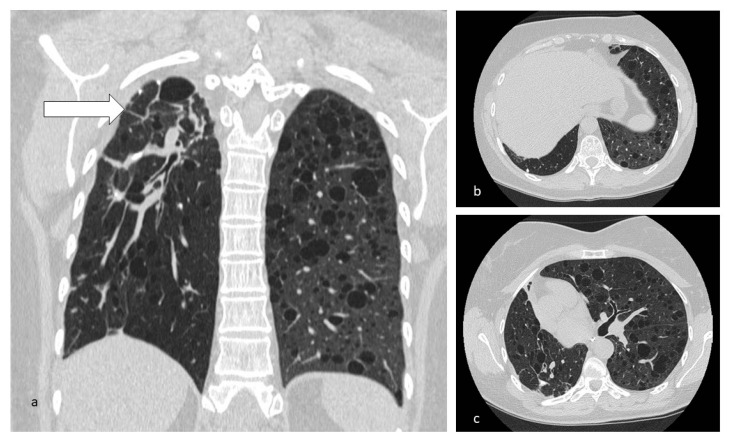
Cysts in LAM typically distributed through all the lungs (**b**,**c**). In this case, the irregularly shaped cystic/fibrotic formation at the right superior lobe is to refer to a previous tubercular infection (**a**).

LAM occurs mostly in women of childbearing age, usually between 17 and 50 years, probably related to the role exercised by the activity of the estrogens, even if cases of prepubertal and octogenarian patients have been also described [16]. Estrogens’ role is unclear, but recent studies demonstrate that estrogenic hormones can activate protein kinase B and favor dysregulated protein translation through up-regulation of Fra1 (Fos-related antigen 1) [17]. In LAM, the development of cysts is due to focal bronchiolar dilation, caused by the proliferation of atypical hamartomatous tumor-like muscle cells in the walls of the airways, which determines a “ball-valve” effect; the progressive amount of cells into the walls narrows the lumen, leading to increased pressure, and then to dilation of bronchioles. LAM cells expand into the interstitium, in the absence of neighboring tissue invasion, but in some cases show airways, the pulmonary artery, diaphragm and retroperitoneal fat involvement, with bronchial cartilage damage, arteriolar wall destruction and pulmonary arteriolar occlusion [18]. LAM cysts are typically rounded in shape, with thin walls (Figure 5), variable diameter (small: 2–5 mm; intermediate: 1–2 cm; large: >2 cm) and ubiquitous distribution (Figure 6 and Figure 7). In later stages of the disease, cysts become larger and sometimes coalescent. Unlike LCH, the costophrenic angles involvement is pathognomonic, and there is sparing of the apical regions [19] (Figure 3); in addition, the adjacent lung parenchyma is normal, although areas of superimposed ground-glass opacities, secondary to alveolar hemorrhage or lymphatic congestion, are frequently detected [20]. Lung nodules are typically absent in LAM, a feature that helps to distinguish it from LCH. The diagnostic criteria—according to the 2016 guidelines—include characteristic HRCT findings plus at least one of the following: [21]:

- Tuberous Sclerosis complex;
- Chylous effusions;
- Angiomyolipomas;
- Lymphatic involvement;
- Serum VEFG-D >800 pg/mL (VEGF-D is a lymphangiogenic growth factor that is increased in the serum of patients with LAM);
- Histological findings.

Frequent symptoms of LAM are dyspnoea, cough and frequent/recurrent pneumothorax (30–40%) or chylothorax, occasionally accompanied by hemoptysis [22]. Lymphatics involvement may be systemic, interesting mediastinal and abdominal regions, where the incidental discovery of renal angiomyolipomas, lymphangioleiomyomas or lymphoadenomegaly is estimated to be at 50%, associated with chylous ascites and chylous effusions [22]. 

### 4.3. Lymphocytic Interstitial Pneumonia (LIP)

LIP represents a benign lymphoproliferative disorder, characterized by the infiltration of the interstitium by lymphocytes, plasma cells and other elements from the lymphoreticular system in the alveolar septa [23]; it is often associated with collagen vascular disorders, such as Sjögren’s syndrome and systemic lupus erythematosus (SLE), and with infectious diseases such as acquired immunodeficiency syndrome (AIDS) [24]; it has been less frequently found in association with Hashimoto’s thyroiditis, pernicious anemia, primary biliary cirrhosis, gravis myasthenia, dysproteinemias, chronic active hepatitis and with Castleman’s disease: in these cases, LIP is considered secondary [25]. LIP predominantly affects patients aged 40–50 years, with a prevalence in the female gender, in the absence of known risk factors [7]. Non-infectious form typically occurs in women in the fifth decade, with coughing and wheezing [16]. Common clinical findings are cough and worsening dyspnoea, and reduction of tolerance to exertion; other symptoms could be fever, chest pain and weight loss, associated with joint pain, characteristic of autoimmune involvement [26]. 

**Figure 8:**
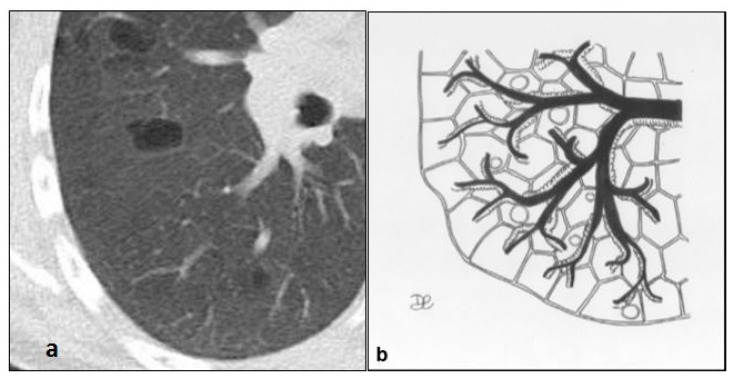
LIP cysts pattern (**a**): multiple cysts, with size ranging from 10 to 30 mm and thin walls, predominantly in peribroncovascular regions (**b**).

**Figure 9:**
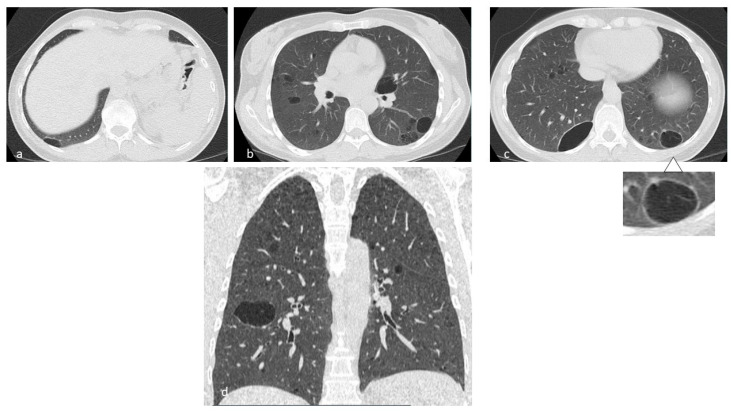
LIP cysts, mostly distributed in peribroncovascular regions (**a**–**d**). Multiplanar reconstructions (MPR) in the coronal plane (**d**).

In LIP, cysts result from dilation of the airways above the stenosis, which is secondary to lymphocytic infiltration of the bronchiolar wall; these formations have thin walls and regular distribution, predominantly in peri-vascular regions, with involvement of the central portion of the lung. The cysts show variable size, ranging from few millimeters to 3 cm [27] (Figure 8 and Figure 9). On chest radiographs, a fine linear or reticular pattern may be found. In the acute phase, CT scan reveals pulmonary cysts and additional findings such as ground-glass opacities with a reticular pattern or small centrilobular nodules with low density, subpleural and peri-lobular nodules with higher density or consolidation [28]. LIP usually responds to steroid or immunosuppressive therapy, so that these opacities may regress in chronic disease; peri-vascular cysts could remain the only residual finding [29]. In some cases, LIP cases complicated with pulmonary fibrosis or that evolved into lymphoma have been described [30]. 

Bronchoalveolar lavage (BAL) allows specific cellular findings of the disease to be detected, such as lymphoid polyclonal proliferation, predominantly CD4+ high-intensity T-cells alveolitis, associated with intra-alveolar macrophages and foci of organizing pneumonia, fibrosis and non-necrotizing granulomas; however, surgical lung biopsy is required for definite diagnosis.

### 4.4. Desquamative Interstitial Pneumonia (DIP)

DIP is an uncommon pulmonary disease caused by abnormal and uniform accumulation and intra-alveolar aggregation of macrophages [31], associated with a few desquamated alveolar epithelial cells in the distal branches of the airways, mild inflammation and fibrosis. The disease occurs in patients aged between 30 and 50 years, with a slight prevalence in the male gender. Rare cases of DIP have been described in children, caused by surfactant gene mutations [32].

Exposure to cigarette smoke is the recognized trigger for manifesting the disease [33]; the most common symptoms include chronic and progressive dyspnoea, dry cough, chest pain and a restrictive pattern in respiratory tests [34]. The prognosis of patients with DIP is very good, because smoking cessation allows an improvement of symptoms; oral steroid therapy may be helpful in patients who continue to smoke. DIP may be observed in association with toxic inhalation, drug reactions, LCH, asbestosis, Gaucher disease and hard-metal pneumoconiosis [35,36].

**Figure 10:**
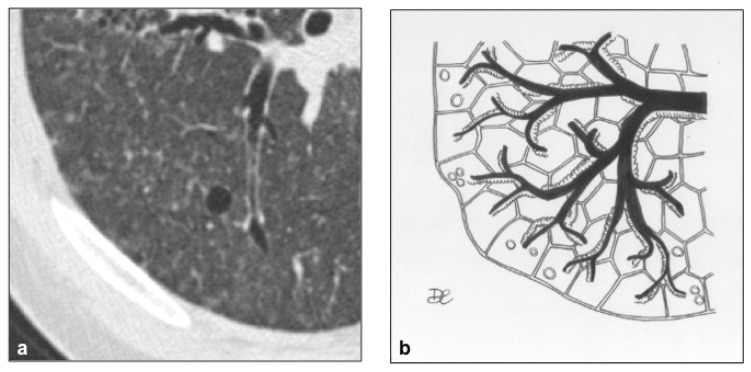
DIP cysts pattern (**a**,**b**). Lung cysts with regular shape, uniform size, thin walls, predominantly localized at the peripheral regions. This figure has been partially modified from ECR 2017/C-2141 Cystic pattern in lung diseases: a simplified HRCT guide based on free-hand drawings, DC Caltabiano, V. Costanzo, L. Mammino, V. Vindigni, S. Torrisi, R. Rosso, LA Mauro, C. Vancheri, S. Palmucci.

**Figure 11:**
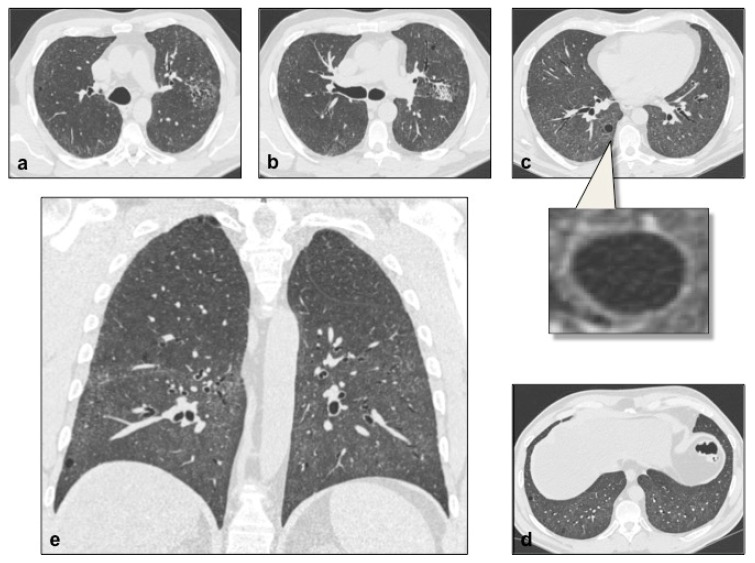
DIP cysts, mostly located in subpleural regions (**a**–**d**). Multiplanar reconstruction (MPR) in the coronal plane (**e**). This figure has been partially modified from ECR 2017 / C-2141 Cystic pattern in lung diseases: a simplified HRCT guide based on free-hand drawings, DC Caltabiano, V. Costanzo, L. Mammino, V. Vindigni, S. Torrisi, R. Rosso, LA Mauro, C. Vancheri, S. Palmucci.

The radiological pattern is characterized by parenchymal areas of “ground glass” attenuation [37], having patchy distribution, predominantly depicted in lower lung zones, peripheral and subpleural regions, where micro-cysts, reticular opacities, irregular lines and traction bronchiectasis can be found [38]; honeycombing—which is considered pathognomonic sign of parenchymal fibrosis—is usually absent [39,40]. Cysts occur typically in lower and peripheral lung zones and involve less than 10% of lungs parenchyma [41] (Figure 10 and Figure 11). Other smoke-induced lung diseases, such as centrilobular emphysema and respiratory bronchiolitis—with patchy air trapping on expiratory scans—may be visible and superimposed to DIP alterations. The small air-filled cysts and ground-glass opacities may be stable or disappear spontaneously with smoking cessation or corticosteroid therapy [42]. Surgical lung biopsy is mandatory to confirm the radiological diagnosis, although BAL and a transbronchial lung biopsy may be helpful; in DIP, BAL normally shows an increased number of macrophages, neutrophils, eosinophils and in rare cases lymphocytes. 

### 4.5. Neurofibromatosis (NF)

NFs are a group of dominant autosomal disorders, affecting about 1 in 3000 individuals [43], characterized by multiple schwannomas on all nerve trunks; the most frequent is NF type 1, so-called Von Recklinghausen disease, associated with gliomas of optic nerves, Lisch nodules, skin changes (such as the cafe-au-lait spots), axillary or inguinal freckling, visceral formations (mediastinal and digestive system tumors), meningeal tumors, bone lesions and scoliosis [44]. Pulmonary involvement occurs in 10%–20% of adult patients with NFs; the typical clinical finding is dyspnoea on exertion, with obstructive or restrictive pattern on pulmonary function tests [45].

**Figure 12:**
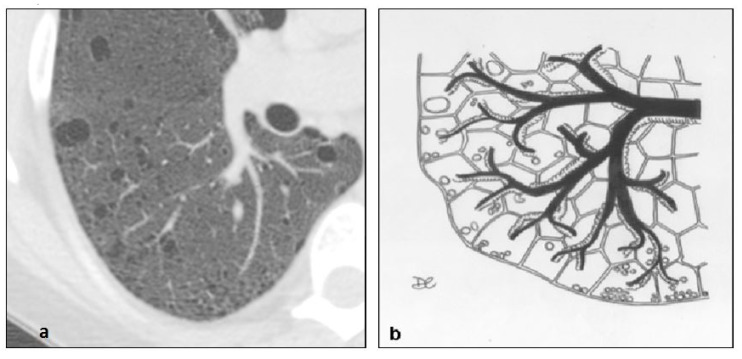
In NF1, the cysts usually present no clearly-defined walls. These lesions are predominant in the superior regions (**a**,**b**). Detail of NF1 cysts (**b**).

**Figure 13:**
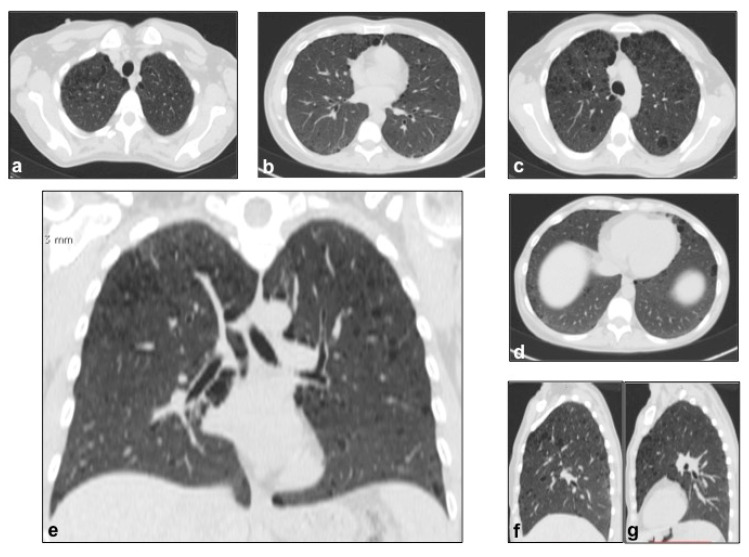
NF1 cysts are diffused and irregularly shaped, with no clearly-defined walls, mainly located in the upper lobes, symmetrically (**a**–**d**). In addition to cysts, ground-glass opacities and emphysema are detected in this case. Multiplanar reconstruction (MPR) in the coronal (**e**) and sagittal (**f**,**g**) planes.

On chest radiographs, a reticular pattern with Kerley’s B lines may be observed in 50% of cases, associated with a typical lung volume increase; on HRCT scans, diffused and irregular shaped lung cysts or bullae can be rarely appreciated, predominantly distributed in upper lobes (Figure 12 and Figure 13), having symmetric location, with a significant risk of pneumothorax [46]; in addition to cysts, basilar bilateral reticulation, “ground glass” opacities and emphysema are often detected in these patients. Other thoracic alterations include cutaneous, subcutaneous and intercostal neurofibromas, “ribbon” ribs, thoracic meningoceles, paraganglioma, pheochromocytoma, intestinal carcinoids, mediastinal masses and pulmonary fibrosis [47].

### 4.6. Birt–Hogg–Dubè (BHD) Syndrome

**Figure 14:**
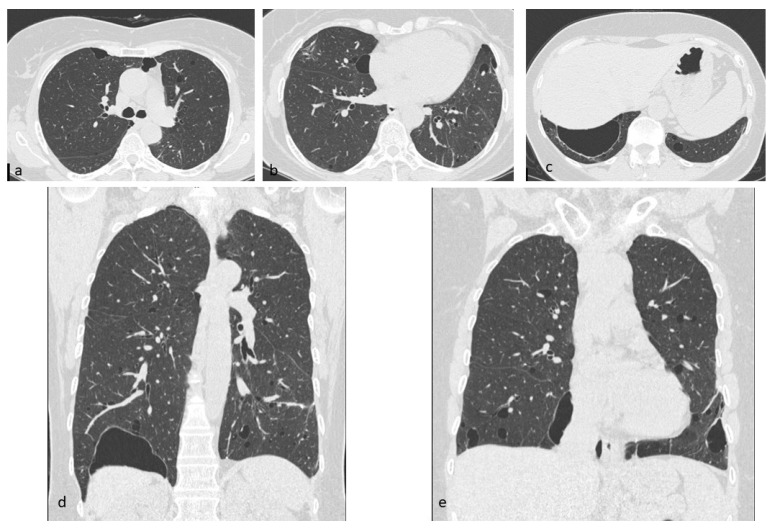
BHD cysts are usually distributed at lung bases (**d**) and in paramediastinal areas (**a**–**c**,**e**). They may have different shape: irregular, septated or round. Multiplanar reconstruction (MPR) in the coronal plane (**d**,**e**).

**Figure 15:**
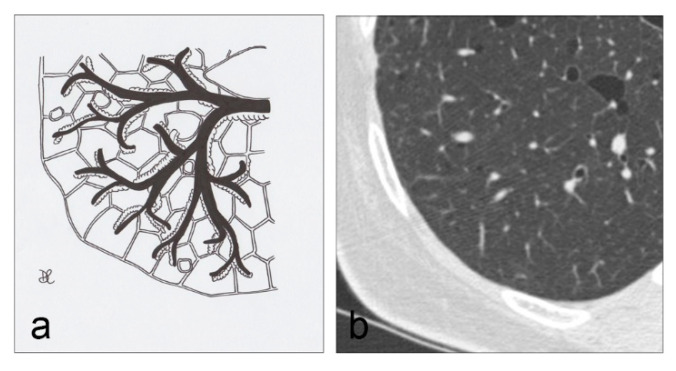
Birt–Hogg–Dubè cyst pattern (**a**,**b**), with multiple thin-walled cysts, usually distributed at lung bases and paramediastinal areas, with different shape: irregular, septated, or round.

BHD is a rare autosomal-dominant systemic disease, which is characterized by the presence of cutaneous lesions—such as fibrofolliculomas, trichiliocosms, acrochordon—mainly located in the head, neck and upper trunk regions; in addition, the disease includes also renal tumors (often multiple and bilateral: oncocytomas and chromophobe carcinomas). This syndrome is associated with mutation of the FLCN gene on chromosome 17p11.2, which encodes for a protein called “folliculin”. Cutaneous lesions, known as fibrofolliculomas, usually appear around the age of 20 years, and manifest as multiple rounded white papules on the nose and cheeks. Other skin alterations include tricodiscomas and acrochordons. The diagnosis is based on clinical data and histological findings of cutaneous lesion; it may be necessary to perform multiple biopsies to ensure the diagnosis. Renal tumors usually manifest at around 50–60 years old; most frequently encountered histotypes are represented by chromofobic renal cells tumor and oncocytic renal cell tumor; clear cell carcinoma and mixed variety carcinoma may be also found. Pulmonary manifestations include multiple thin-walled cysts, usually distributed at lung bases and paramediastinal areas (Figure 14 and Figure 15). They may have different shapes: irregular, septated or round [48,49]. Cysts are usually circumscribed by normal aerated parenchyma.

### 4.7. Infectious Pneumatocele, Recurrent Respiratory Papillomatosis (RRP) and Paragonimiasis

**Figure 16:**
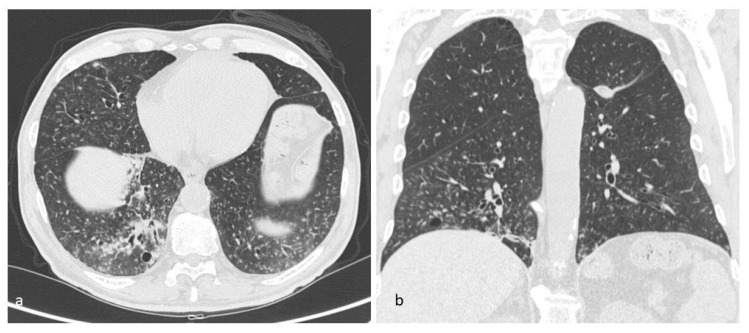
Pneumatocele in a patient with known infection from *Pneumocystis carinii*. Multiplanar reconstruction (MPR) in the coronal plane (**b**). A thin-walled cystic air space is located in the lateral-basal segment of the lower right lobe (**a**).

The pneumatocele is a cyst-like lesion of the pulmonary parenchyma, thin-walled and air-filled, which is usually found in acute pneumonias—mainly caused by *Streptococcus pneumoniae*, *E. coli*, *Klebsiella*, *Staphylococcus* and non-bacterial organisms (such as *Pneumocystis jirovecii* and Coccidiomycosis pneumonia) (Figure 16) [50]; it develops after trauma or inhalation of hydrocarbon fluids [51]. In the acute phase, the pneumatocele has thick walls, whereas in the chronic phase the walls become thinner, in contrast to abscesses or pulmonary gangrenes. Cysts caused by *Pneumocystis jirovecii* infections are normally numerous, bilateral, usually located in the upper lobes, having variable size and wall thickness. The pathogenetic mechanism is based on parenchymal necrosis and airways valve obstruction [52]. The development of a post-traumatic pneumatocele is generally preceded by the laceration of the lung parenchyma [51,53].

Recurrent respiratory papillomatosis (RRP) is a pediatric disorder, caused by the human papillomavirus [54], which typically involves the upper respiratory airways determining nodules and obstruction [55]. The extension of papillomas to bronchi and bronchioles is rare, and it may be superimposed to the cystic lung pattern, due to multiple cavitations; these cysts are thin-walled, irregularly shaped, air-filled, less than 5 cm in diameter, located in the lower zones of the lung [56].

Paragonimiasis is a parasitic zoonosis, caused by *Paragonimus westermani*, and it is acquired by eating crustaceans. Pulmonary findings include ill-defined nodules, areas of consolidations, linear opacities and cysts, resulting from arteriolar and venous occlusion, determining ischemic infarctions [57]; cysts present size from 5 to 15 mm in diameter, and have an irregular wall thickness [58].

### 4.8. Cystic Fibrosis (CF)

Cystic fibrosis (CF) is a multisystemic autosomal recessive disorder related to alteration of the structure of the CF transmembrane conductance regulator (CFTR) [59]. It is characterized by an anomalous sodium and chloride transport through the epithelium in all exocrine tissues, resulting in viscous secretions in the pulmonary, pancreatic, hepatic, intestinal and genital tract and determining an increased incidence of bacterial airways infections [60]. The main clinical findings in child patients affected by CF are chronic and recurrent infections, associated with abnormal sweat chlorides. 

**Figure 17:**
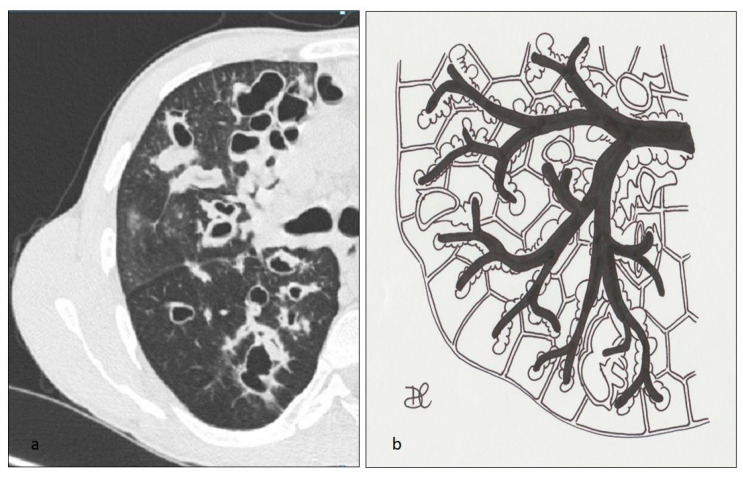
Cystic fibrosis (CF) pattern (**a**), multiple cystic lesions, represented by cystic bronchiectasis, filled by mucous secretion, abscess cavities or bullae, mainly located in subpleural and upper zones of the lung; these bronchiectases may be variable in size, with regular shape, wide distribution and thickened walls (**b**).

**Figure 18:**
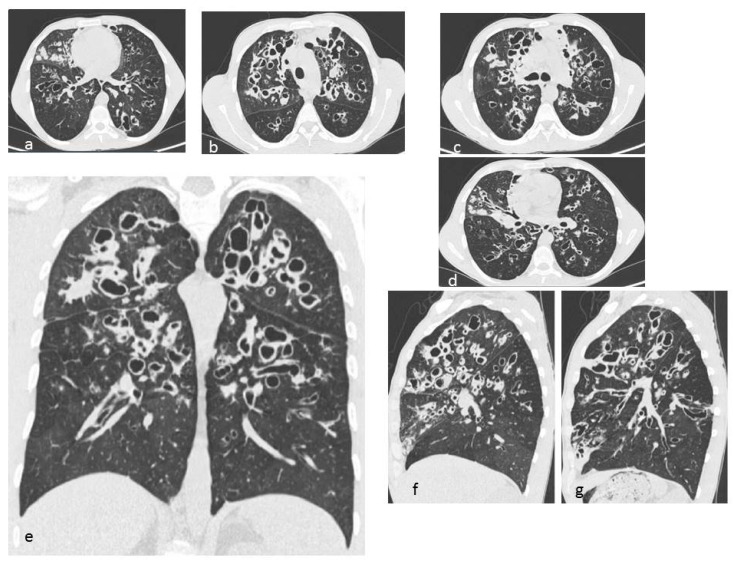
CF, multiple cystic bronchiectases, filled by mucous secretion, abscess cavities or bullae, mainly located in subpleural and middle and upper lung regions (**a**–**d**). Multiplanar reconstruction (MPR) in the coronal (**e**) and sagittal planes (**f**,**g**).

Radiologically, lung involvement consists of multiple cystic lesions—due to cystic bronchiectases, filled by mucous secretion—associated with the development of abscess cavities or bullae, mainly located in subpleural and upper zones of lungs [61]; in addition, tree-in-bud pattern with mosaic perfusion and focal air-trapping areas on expiratory scans may be depicted, due to the presence of an obstructive disease of small airways [62] (Figure 17 and Figure 18). Mediastinal lymphatic nodes enlargement, pleural thickening and pulmonary artery dilatation may be also appreciated on HRCT images.

### 4.9. Aging Lung Cysts

**Figure 19:**
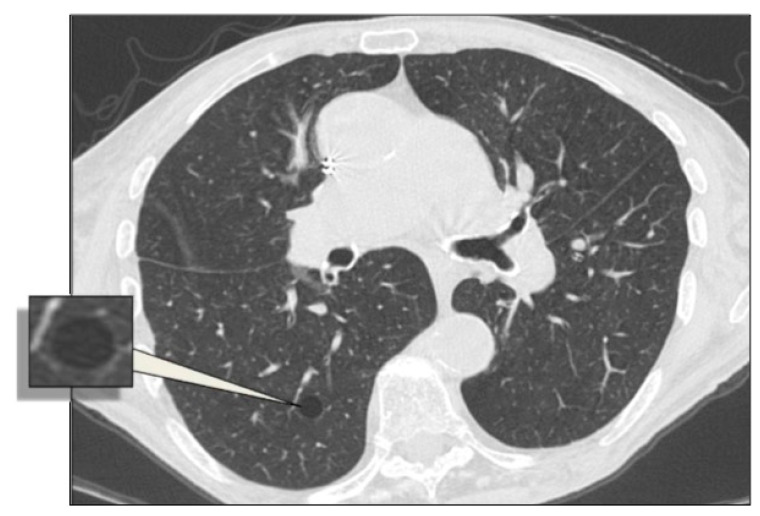
Aging lung manifests with the progressive air space enlargement. These cysts are thin-walled, and have a wide distribution. This figure has been partially modified from ECR 2017/C-2141 Cystic pattern in lung diseases: a simplified HRCT guide based on free-hand drawings, DC Caltabiano, V. Costanzo, L. Mammino, V. Vindigni, S. Torrisi, R. Rosso, LA Mauro, C. Vancheri, S. Palmucci.

**Figure 20:**
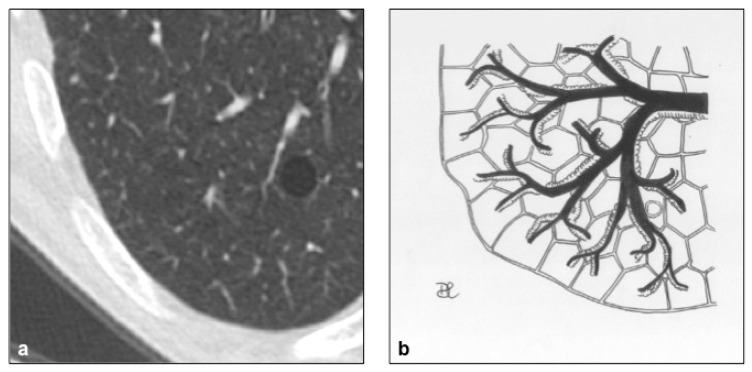
Aging lung cysts pattern (**a**,**b**). These cysts can be found in any portion of the lungs; they usually show rounded shape and they are limited by thin walls. This figure has been partially modified from ECR 2017/C-2141 Cystic pattern in lung diseases: a simplified HRCT guide based on free-hand drawings, DC Caltabiano, V. Costanzo, L. Mammino, V. Vindigni, S. Torrisi, R. Rosso, LA Mauro, C. Vancheri, S. Palmucci.

The elderly lung is characterized by the progressive air space enlargement. Various studies have demonstrated a high prevalence of thin-walled cysts in many elderly individuals (25% in 75-year-old patients, absent in patients under 55 years old) [63] (Figure 19 and Figure 20); these cysts have a wide distribution and they do not show any correlation with cigarette smoking.

### 4.10. Cystic Metastasis

Cystic lung metastasis is most frequently found in patients with angiosarcoma or squamous cell carcinoma, mainly of the head and neck. As for the formation of the cysts, four mechanisms have been detected: first, the excavation of a nodule; second, the infiltration of malignant cells into a previously existing bulla can hesitate into a cyst formation; third, cancer cells infiltrate into alveolar walls, determining a ball-valve effect and cystic dilation; fourth, malignant cells proliferation can evolve in blood-filled cystic air spaces [64]. Multiple and thin-walled cysts, multiple solid nodules and hemorrhagic lesions are the most common findings in angiosarcoma. The cysts, which are mainly located at the basilar zones, may present air-fluid levels and they may show different sizes [65]. 

### 4.11. Amyloidosis 

Amyloidosis is a rare disease, characterized by the extracellular deposition of abnormal proteins [66,67]. Typically, the amyloid protein deposit shows green birefringence with Congo red stain under polarized light [67]. Pulmonary involvement in amyloidosis is rare [67], and it can manifest with cystic lung disease. Amyloid-associated cystic lung disease can occur with Sjögren’s syndrome and mucosa-associated lymphoid tissue lymphoma [68]. The pathogenetic mechanism that leads to cysts development is due to the narrowing of the airways from inflammation or amyloid protein deposits and results in a ball-valve effect. Alternatively, amyloid deposition may cause capillary disruption, alveolar wall destruction and eventually cysts formation. At HRCT scans, lung cysts are numerous and they have peribronchovascular or subpleural distribution; moreover, they are frequently associated with nodular lesions and calcifications. Cysts can be round or lobulated, with variable size, and thin-walled. Other associated findings include interlobular septal thickening, ground-glass opacity, honeycombing, circumferential thickening of the tracheal wall and lymphadenopathy [68].

## 5. Conclusions

Given their large radiological heterogeneity, a correct description of the features of cystic diseases is essential to achieve a correct diagnosis, although radiologists should never underestimate the concomitant parenchymal abnormalities. Therefore, HRCT represents an indispensable diagnostic tool to define the correct diagnosis, improving patient treatment and outcome.

## Figures and Tables

**Table 1:** The main high-resolution computed tomography (HRCT) features, wall thickness (thick or thin cut-off: 2 mm) and distribution of cysts, plus associated features in Langerhans cell histiocytosis (LCH), lymphangioleiomyomatosis (LAM), lymphocytic interstitial pneumonitis (LIP), desquamative interstitial pneumonitis (DIP), neurofibromatosis (NF1), Birt–Hogg–Dubè (BHD) syndrome, pneumatoceles, cystic fibrosis and aging lung cysts are described.

	Disease	Hrct Features	Wall	Distribution	Other Features
**Diffused**	LCH	Variable shape, “bizzarre” variable size Thin walls	thick	Wide spreading, with sparing of costo-phrenic angles	Centrilobular nodules at early stages
	LAM	Round and regular shape Uniform size (10–20 mm) Thick walls	thin	Wide spreading No zonal sparing	No nodules
	LIP	Regular shape Uniform size Thin walls	thin	Peri-broncho-vascular regions	Ground-glass opacities, septa thickening, centrilobular nodules
	DIP	Regular shape Uniform size Thin walls	thin	Peripheral regions	Ground-glass opacities, linear opacities
	BHD	Variable shape, irregular, septated or round	thin	Lung bases paramediastinal areas	Pneumothorax
**Other**	NF1	Irregular shape and small size	thick	Predominant in the upper lobes	Ground-glass areas with reticular basal opacities
	Pneumatocele	Regular shape and variable size	thick/thin	Focal or multi-focal predominant at upper lobes	Ground-glass opacities in peri-hilar regions
	Cystic Fibrosis	Regular shape and variable size bronchiectasis	thick	Predominant in upper lobes and dorsal segments of lower lobes	Air trapping areas, impaired perfusions areas, enlarged mediastinal and hylar lymphnodes
	Aging Lung Cysts	Regular shape and variable size	thin	Wide	Linear irregular opacities and reticular subpleural opacities

## Supplementary Materials

The following are available online at https://www.mdpi.com/2075-4418/10/6/346/s1.
