# Supplementary material for: Cystic Interstitial Lung Diseases: A Pictorial Review and a Practical Guide for the Radiologist
Source: Diagnostics (Basel). 2020 May 27;10(6):346. doi: 10.3390/diagnostics10060346 (PMC7345690; doi:10.3390/diagnostics10060346)
Supplement: Supplementary file 1 [file diagnostics-10-00346-s001.zip › flowchart.docx]

Articles excluded for the following reasons:

Full text not available; non-English articles; irrelevant articles; articles not related to the topic; articles from the same author (n= 281)

Articles obtained from reference lists of retrieved articles (n= 38)

Included full-text articles

(n= 68)

Articles screened for title and abstract (n=311)

Literature search from the PubMed database (from 1966 to 2020)
