# Supplementary material for: Cystic Interstitial Lung Diseases: A Pictorial Review and a Practical Guide for the Radiologist
Source: Diagnostics (Basel). 2020 May 27;10(6):346. doi: 10.3390/diagnostics10060346 (PMC7345690; doi:10.3390/diagnostics10060346)
Supplement: Supplementary file 1 [file diagnostics-10-00346-s001.zip › Table1word.docx]

|  | **DISEASE** | **HRCT FEATURES** | **WALL** | **DISTRIBUTION** | **OTHER FEATURES** |
| --- | --- | --- | --- | --- | --- |
| **DIFFUSED** | **LCH** | Variable shape,  “bizzarre” variable size  Thin walls | thick | Wide spreading, with sparing of costo-phrenic angles | Centrilobular nodules at early stages |
|  | **LAM** | Round and regular shape  Uniform size (10-20 mm)  Thick walls | thin | Wide spreading  No zonal sparing | No nodules |
|  | **LIP** | Regular shape  Uniform size  Thin walls | thin | Peri-broncho-vascular regions | Ground-glass opacities, septa thickening, centrilobular nodules |
|  | **DIP** | Regular shape  Uniform size  Thin walls | thin | Peripheral regions | Ground-glass opacities, linear opacities |
|  | **BHD** | Variable shape, irregular,  septated or round | thin | Lung bases paramediastinal areas | Pneumothorax |
| **OTHER** | **NF1** | Irregular shape and small size | thick | Predominant in the upper lobes | Ground-glass areas with reticular basal opacities |
|  | **PNEUMATOCELE** | Regular shape and variable size | thick/thin | Focal or multi-focal  predominant at upper lobes | Ground-glass opacities in peri-hilar regions |
|  | **CYSTIC FIBROSIS** | Regular shape and variable size bronchiectasis | thick | Predominant in upper lobes and dorsal segments of lower lobes | Air trapping areas, impaired perfusions areas, enlarged mediastinal and hylar lymphnodes |
|  | **AGING LUNG CYSTS** | Regular shape and variable size | thin | Wide | Linear irregular opacities and reticular subpleural opacities |
