# Supplementary figures and images for: Cystic Interstitial Lung Diseases: A Pictorial Review and a Practical Guide for the Radiologist
Source: Diagnostics (Basel). 2020 May 27;10(6):346. doi: 10.3390/diagnostics10060346 (PMC7345690; doi:10.3390/diagnostics10060346)

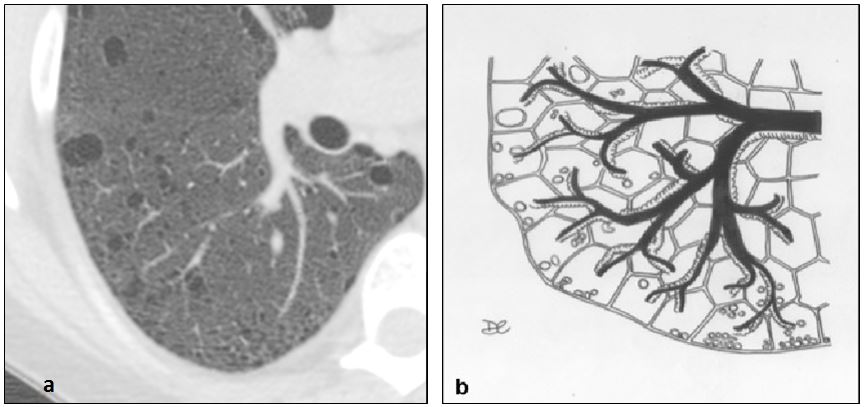

Supplement: Supplementary file 1 [file diagnostics-10-00346-s001.zip › FIG12NEW.JPG]

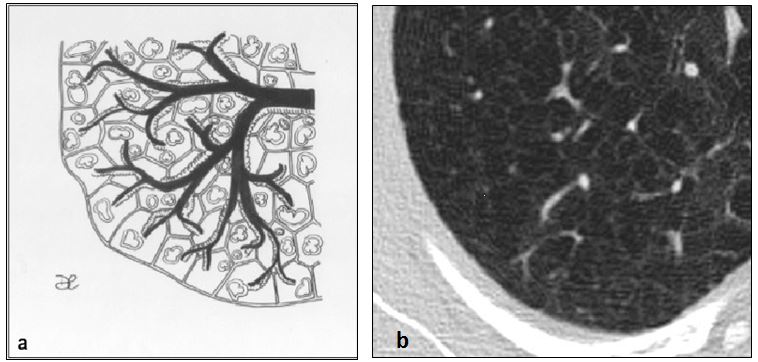

Supplement: Supplementary file 1 [file diagnostics-10-00346-s001.zip › FIG2NEW.JPG]

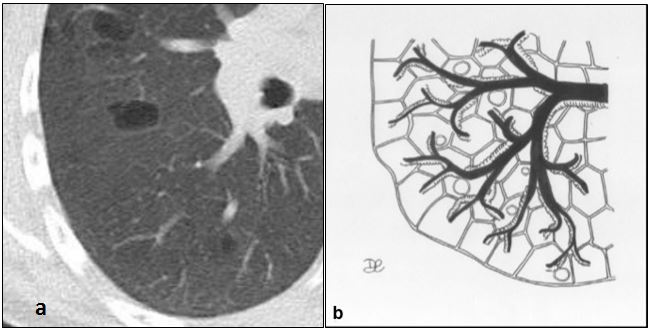

Supplement: Supplementary file 1 [file diagnostics-10-00346-s001.zip › FIG8NEW.JPG]

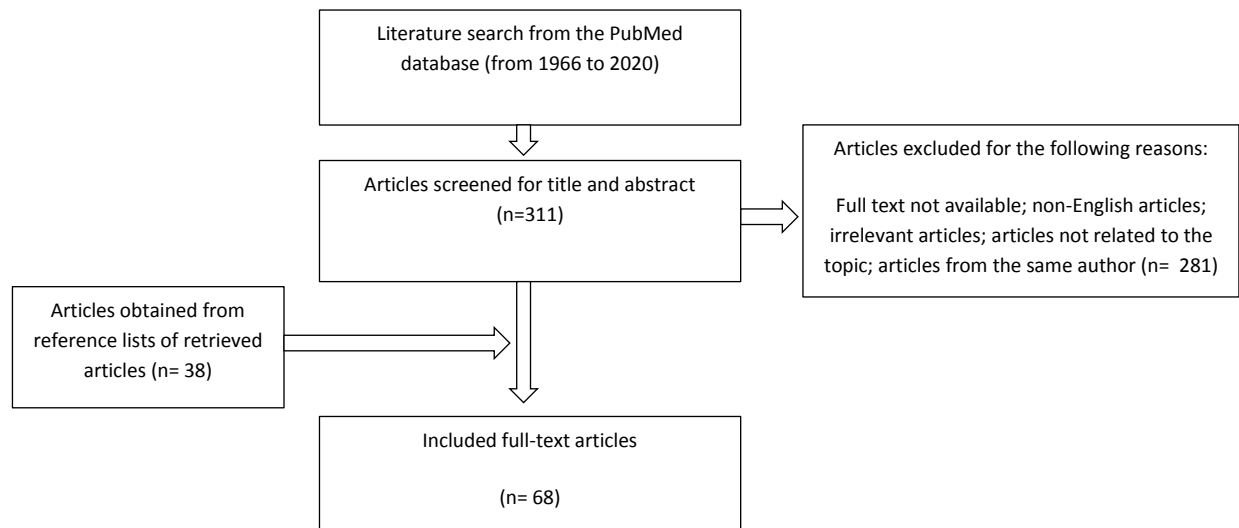

Supplement: Supplementary file 1 [file diagnostics-10-00346-s001.zip › flowchart.pdf]
